# Hypothyroidism after radiation exposure: brief narrative review

**DOI:** 10.1007/s00702-020-02260-5

**Published:** 2020-10-09

**Authors:** Christoph Reiners, Valentina Drozd, Shunichi Yamashita

**Affiliations:** 1 Department of Nuclear Medicine, University Hospital, Oberduerbacherstr.6, 97080 Wuerzburg, Germany; 2 International Foundation Arnica, Minsk, Belarus; 3 Global Exchange Center, Fukushima Medical University, Fukushima, Japan; 4 Center for Advanced Radiation Emergency Medicine, National Institutes for Quantum and Radiological Science and Technology, Chiba, Japan

**Keywords:** Thyroid, Hypothyroidism, Autoimmune thyroiditis, Diagnostic medical radiation exposure, Therapeutic medical radiation exposure (EBRT/ RAI), Other radiation exposure (atomic bombing/nuclear accidents)

## Abstract

The thyroid gland is among the organs at the greatest risk of cancer from ionizing radiation. Epidemiological evidence from survivors of radiation therapy, atomic bombing, and the Chernobyl reactor accident, clearly shows that radiation exposure in childhood can cause thyroid cancer and benign thyroid nodules. Radiation exposure also may induce hypothyroidism and autoimmune reactions against the thyroid, but these effects are less well-documented. The literature includes only a few, methodologically weak animal studies regarding genetic/molecular mechanisms underlying hypothyroidism and thyroid autoimmunity after radiation exposure. Rather, evidence about radiation-induced hypothyroidism and thyroid autoimmunity derives mainly from follow-up studies in patients treated with external beam radiotherapy (EBRT) or iodine-131, and from epidemiological studies in the atomic bombing or nuclear accident survivors. Historically, hypothyroidism after external irradiation of the thyroid in adulthood was considered not to develop below a 10–20 Gy dose threshold. Newer data suggest a 10 Gy threshold after EBRT. By contrast, data from patients after iodine-131 “internal radiation therapy” of Graves´ disease indicate that hypothyroidism rarely occurs below thyroid doses of 50 Gy. Studies in children affected by the Chernobyl accident indicate that the dose threshold for hypothyroidism may be considerably lower, 3–5 Gy, aligning with observations in A-bomb survivors exposed as children. The reasons for these dose differences in radiosensitivity are not fully understood. Other important questions about the development of hypothyroidism after radiation exposure e.g., in utero, about the interaction between autoimmunity and hypofunction, and about the different effects of internal and external irradiation still must be answered.

## Introduction

Hypothyroidism is defined as a state of thyroid hormone deficiency and diagnosed by blood levels of thyroid-stimulating hormone (TSH) elevated above the upper limit of the reference value; 4 mU/L is the most frequently applied threshold. Moderately-elevated TSH levels (not higher than ca. 10 mU/L) usually do not cause clinical symptoms of hypothyroidism, since under those conditions, the thyroid mostly can maintain normal blood levels of the thyroid hormones thyroxine (T4) and triiodothyronine (T3). This compensated state, therefore, is a biochemical diagnosis only and is called “subclinical” or “occult” hypothyroidism. However, according to a meta-analysis of data from more than 55,000 adults, undiagnosed, untreated subclinical hypothyroidism is associated with an increased risk of coronary heart disease and related mortality, especially in elderly patients (Rondon et al. 2010). At higher TSH levels, and with T4 and T3 levels below reference ranges, clinical symptoms of “clinical” or “overt” hypothyroidism will develop, e.g., weight gain, cold intolerance, obstipation, edema, dry skin, bradycardia, and fatigue. According to a recent meta-anlaysis, autoimmune thyroiditis as a frequent cause of subclinical or overt hypothyroidism is associated with depression and anxiety disorders (Siegmann et al. 2018).

In the US National Health and Nutrition Examination Survey III, conducted from 1988–1994, 4.3% of the 13,444 members of the “healthy” population who were tested were subclinically hypothyroid, and 0.3%, clinically hypothyroid. The incidence of both forms of hypothyroidism was higher in females and increased with age. In the same survey, measurable autoantibodies against thyroid peroxidase (TPOAb) were found in 8.0% of males and 14.6% of females (Hollowell et al. 2002).

Compared to the US, where the dietary iodine supply is sufficient, Germany is still a country with moderate iodine deficiency. A 2008–2012 study of the prevalence of thyroid disorders in Northeast Germany (Khattak et al. 2016) investigated a 4420-subject representative sample of the adult general population and revealed subclinical or clinical hypothyroidism in 2.4% of males and 3.6% of females; TPOAb were detected in 3.7% of males and 7.1% of females.

The thyroid gland is one of the organs that are most sensitive to, and at highest risk of cancer from ionizing radiation. There is clear evidence from medically-irradiated patients, survivors of the atomic bombing, and people affected by the Chernobyl reactor accident, that radiation exposure in childhood can cause thyroid cancer and benign thyroid nodules later in life (Ron and Brenner 2010; UNSCEAR 2011; Imaizumi et al. 2017). Apart from that, autoimmune reactions involving the thyroid, thyroid atrophy, and hypothyroidism, may be induced by radiation exposure (Riley et al. 2019). However, these radiation effects are not as well-documented as are radiation-induced cancer and nodular disease of the thyroid. Moreover, relevant data tend to be dispersed widely throughout the literature, due to the large variety of forms of radiation exposure.

We thus decided to develop a narrative review focusing on the topic. In addition to publications with which the authors already were familiar, this review was based on literature identified through a search of the PubMed database for publications in English or German. The keywords hypothyroidism, autoimmune thyroiditis, radiation, diagnostic medical radiation exposure, therapeutic medical radiation exposure (EBRT/RAI), and other radiation exposure (atomic bombing/nuclear accidents) were used, each alone and in various combinations.

We begin the review with a discussion of mechanisms responsible for the induction of radiation-induced hypothyroidism. We then examine findings regarding incidence of hypothyroidism and/or thyroid auto-immunity in different irradiated populations, and dose thresholds, if any, for development of these conditions. We conclude by focusing on unanswered questions regarding these somewhat lesser-known effects of radiation exposure in childhood.

The primary objective of this review is to inform radiation protection experts, general practitioners, and the public about hypothyroidism and autoimmune thyroiditis as possible risks of therapeutic medical radiation exposure and radiation emergencies.

## Hypothyroidism after thyroid irradiation: mechanisms

### Autoimmune thyroiditis

The most frequent cause of non-radiation induced hypothyroidism is autoimmune thyroiditis. Apart from deterministic effects such as necrosis and apoptosis, radiation exposure can cause autoimmune processes in which autoantibodies are produced against cellular components of thyrocytes. The autoantibodies’ binding to their target antibody-dependent cell-mediated cytotoxicity, which may lead to intra-thyroidal inflammation with subsequent hypothyroidism. This process is not fully understood (Riley et al. 2019), but its nature may be stochastic, not limited to threshold radiation doses (Nagayama 2018). On the other hand, relatively high radiation doses may induce autoimmune hyperthyroidism too; this complication is well-known but rare (appr.1%) in the radioiodine treatment of autonomous hyperfunctioning thyroid nodules (Dunkelmann 2004). In these cases, TSH receptor-stimulating antibodies become prevalent, instead of antibodies that induce destruction of thyrocyte components (Nagayama 2018).

### Thyroid atrophy

According to the International Classification of Diseases, 10th edition (WHO 2016), besides autoimmune thyroiditis (E.06.3), another cause of hypothyroidism (E3.09) is atrophy of the thyroid (E3.04). Atrophy of the thyroid and subsequent hypothyroidism is frequently observed in patients with Graves´ disease (autoimmune hyperthyroidism) or thyroid cancer as an obvious consequence of surgical resection of considerable portions of the thyroid ([near-] total thyroidectomy) with or without widespread destruction of thyroid tissue by radioiodine (iodine-131 [I-131])(Chaker et al. 2017; Riley et al. 2019). Historically, other causes of thyroid atrophy have been rare; however, recently, thyroid atrophy has been described as a frequent side effect in a considerable proportion (ca. 50%) of patients treated with the “targeted” anti-cancer drug sunitinib (Shinohara et al. 2011).

Regarding radiation-induced thyroid atrophy, deterministic radiation effects (tissue reactions) usually appear shortly after exposure and are characterized by a dose threshold and a clear dose–effect association. Above the threshold, cells and tissues tend to be more frequently and severely damaged, and to more often lose their function as the radiation dose increases. In a recent comprehensive review about the effects of I-131 radioiodine treatment of the diseased thyroid gland, Riley et al. (2019) summarized, “I-131 induces cell death both by direct and indirect damage to thyrocyte DNA largely through apoptosis. Some studies, however, have described detailed findings of cell death by necrosis following I-131”. In this sense, thyroid atrophy with subsequent hypothyroidism is a typical deterministic radiation effect.

## Incidence of hypothyroidism and/or thyroid auto-immunity in various populations after radiation exposure

The highest radiation doses to the thyroid result either from treatment with radioidine I-131 (RAI) in nuclear medicine (up to 300–400 Gy) or EBRT with percutaneous irradiation (up to 70–80 Gy). By comparison, thyroid doses resulting from radiation exposure from atomic bombs or nuclear accidents reached 4 Gy at maximum and not more than 0.5 Gy in ca. 90% of the people exposed (Ron & Brenner 2010, UNSCEAR, Imaizumi et al. 2017). In contrast, thyroid doses involved with radiological imaging are comparatively low, e.g., 10–20 mGy from computed tomography (CT) of the neck or the chest (Su et al. 2014).

### Radioiodine therapy

Bonnema and Hegedüs systematically reviewed the literature on “Radioiodine therapy in benign thyroid diseases: effects, side effects and factors affecting therapeutic outcome” (Bonnema and Hegedüs 2012). Regarding hypothyroidism in patients treated with RAI for immunogenic hyperthyroidism (Graves´ disease), the effect/time curve is exponential, with a steep increase after the first year post-I-131 (24%); 59% of patients become hypothyroid after 10 years, and 85%, after 25 years (Metso et al. 2004). This curve is less steep and more linear in patients with non-immunogenic hyperthyroidism (“toxic” nodules or goiter), with the percentage of hypothyroid patients 4% after 1 year, 15% after 10 years, and 32% after 25 years (Metso et al. 2004).

A drawback of most published studies about the outcome of RAI in patients with benign thyroid diseases is that the therapeutic activity of I-131 (in MBq) is mostly dosed empirically. The activity is determined considering thyroid mass only, not taking into account important individual variables of radioiodine kinetics such as maximum uptake and effective half-time (Ron & Brenner 2010). Only if all three of the above variables are taken into account, is it possible to apply the Marinelli formula for pretherapeutic dosimetry

$${\text{activity}} = \frac{{{\text{dose}}\;{\text{(Gy)}} \times {\text{thyroidmass}}\,{\text{(g)}} \times 22.5}}{{{\max}{.}\;{\text{iodine}}\;{\text{uptake}}\;{\text{(\% )}} \times {\text{effective}}\;{\text{half } - \text{ lifetime}}\;{\text{(day)}}}}$$

**Fig. 1:**
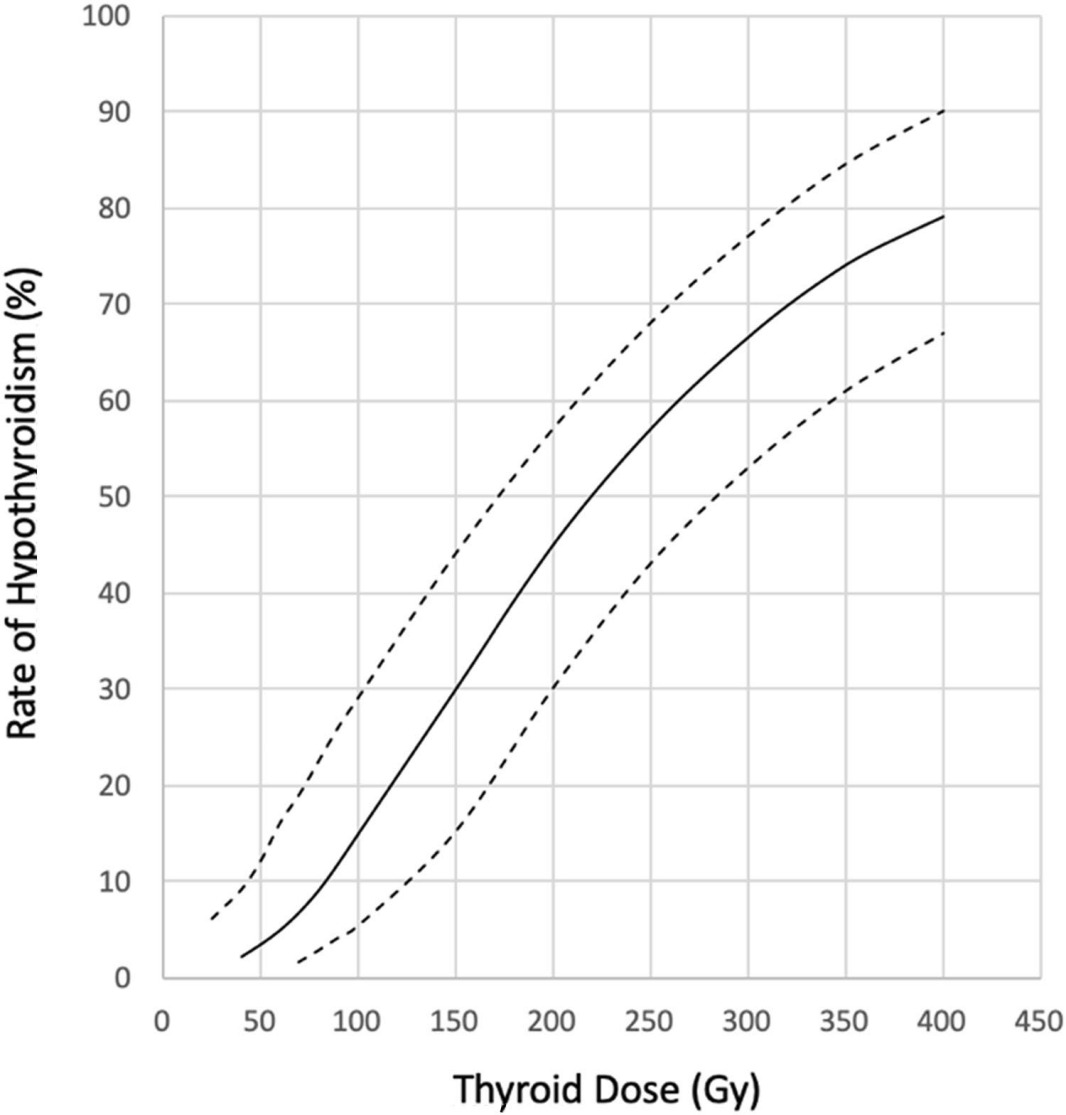
Dose–response rate curves (solid line: mean, dotted lines: 95% confidence interval) for hypothyroidism with increasing thyroid doses after RAI in patients with Graves’ disease (authors’ data from a German multicenter study, reconstructed after Peters et al. 1995)

This formula allows calculation of an I-131 activity (MBq) likely to achieve a target radiation dose to the thyroid, which is usually 300 or 400 Gy in patients with Graves´ disease (Hänscheid et al. 2013). In a German prospective multicenter study, the empiric and the dosimetric approaches were compared in 205 patients, with better results regarding concerning euthyroidism after RAI for the latter strategy. Most importantly, individual dosimetry performed after I-131 therapy allowed the most exact documentation possible of the radiation dose that actually resulted from RAI (Peters et al.^1997^). Figure 1 shows that after 6 months after RAI of Graves’ disease, no statistically significant effect regarding induction of hypothyroidism was observed for thyroid doses below 50 Gy; the 50% intercept for the rate of hypothyroidism corresponded to a thyroid dose of appr. 220 Gy, and 80% of patients became hypothyroid after receiving a dose of 400 Gy (Peters et al. 1997).

### External beam radiotherapy

If external beam radiation therapy (EBRT) of the cervical region is used to treat head and neck cancers, lymphomas, or malignancies of the central nervous system, exposure of non-target tissues, e.g., the thyroid, cannot be prevented. Hypothyroidism can, therefore, develop months to years after EBRT (Rønjom et al. 2015). In a prospective study from Denmark involving 203 patients with head and neck cancer, the estimated five-year incidence of EBRT-induced hypothyroidism was 26%. The median thyroid dose amounted to 40 Gy (minimum–maximum:1.2–68.0 Gy). Applying and testing a Normal Tissue Complication Probability Model, significant risk factors for the development of hypothyroidism were a small thyroid volume and a high mean radiation dose to the gland (Rønjom 2016).

**Fig. 2:**
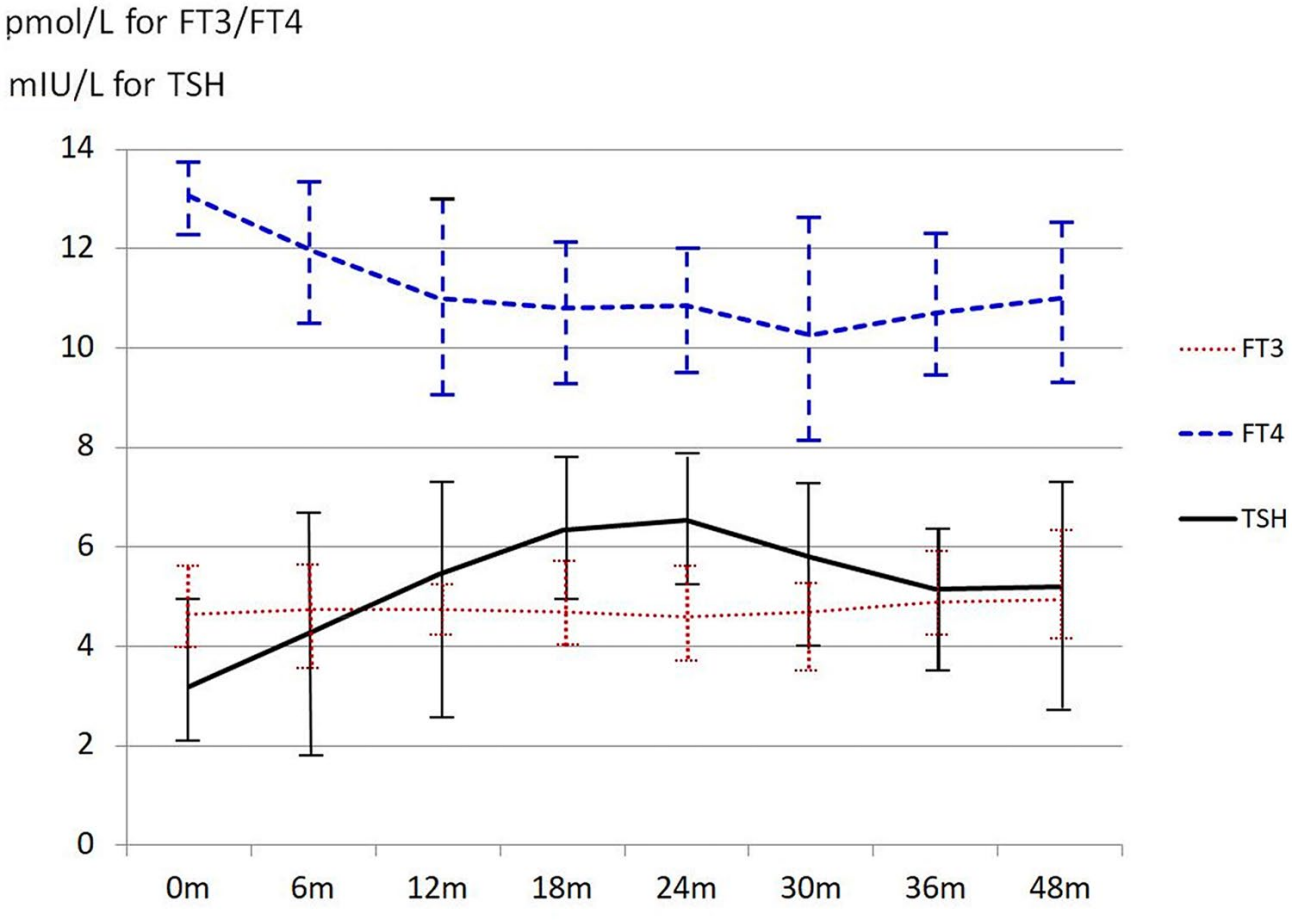
Mean TSH and mean free thyroid hormone levels from pre-radiotherapy to 48 months post-EBRT in patients with nasopharyngeal cancer (*N* = 68). The error bars represent the standard deviation (SD) of the hormone levels at the given measurement time. *0m* pre-radiotherapy, *FT3* free triiodothyronine, *FT4* free thyroxine, *m* month, *TSH* thyroid-stimulating hormone (Lin et al. 2018, reproduced with permission under the PLOSone Creative Common Attributive Licence)

Lin et al. recently published a prospective observational study of 68 patients with nasopharyngeal cancer from pre-radiotherapy to 48 months post-EBRT (Lin et al. 2018). Figure 2 shows that in their study, mean TSH levels increased shortly after radiotherapy, peaked at 18–24 months, and then decreased, albeit remaining elevated above an upper level of normal of 4.0 U/L.

**Fig. 3:**
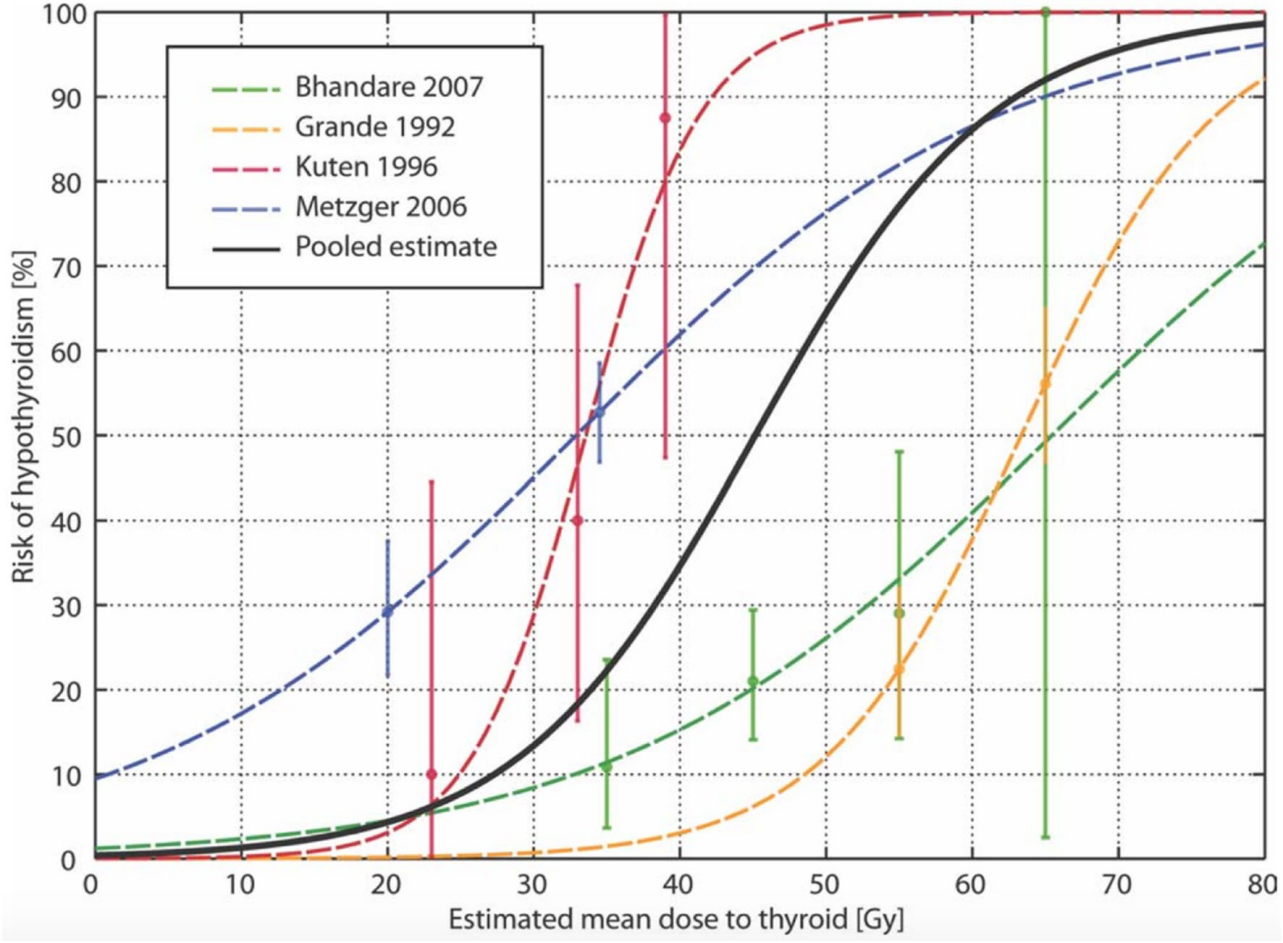
Dose–response analysis (blue, red, green, and yellow broken lines) and the overall pooled estimate (solid black line) of 4 studies of the incidence of hypothyroidism in patients receiving EBRT in the neck region for cancer, based on meta-analysis of the steepness of the curve and of the dose to the thyroid resulting in a 50% rate of hypothyroidism in the pooled estimate of 4 studies (Vogelius et al. 2011, with permission)

Vogelius et al. (2011) reviewed risk factors for hypothyroidism in a meta-analysis involving patients with a variety of malignant diseases needing EBRT of the neck region. Figure 3 shows the original dose–response curves from each of 4 studies and the overall pooled estimate (in black). This pooled curve suggests that hypothyroidism rarely occurs at doses < 10 Gy; the 50% intercept of the curve corresponds to 45 Gy. However, there was marked heterogeneity among studies (see Fig. 3), the most likely cause being differences in follow-up time and intensity.

Inskip et al. (2018) recently published a report about the incidence of both overt and subclinical hypothyroidism in a large cohort of childhood cancer survivors (*N* = 12,015) who had been treated with EBRT of the neck, the base of the skull, or both. The cumulative proportion affected with hypothyroidism was highest in patients irradiated for Hodgkin´s disease (32.3%; 95% confidence interval [CI]: 29.5%–34.9%, *n* = 1550) and cancers of the central nervous system (17.7%; 95% CI: 15.2–20.4%, *n* = 1516). The incidence was significantly associated with the radiation dose to the thyroid and the pituitary. Risks of EBRT-induced hypothyroidism were inversely associated with age at exposure and remained elevated for more than 25 years after exposure.

### Medical diagnostics

Relative to planar radiography, CT scans of the head and neck region may expose the thyroid to comparably high doses of up to 10–20 mGy in adults, as noted earlier (Sinnott et al. 2010; Su et al. 2014). Doses in children are higher, up to 30–50 mGy (Spampinato et al. 2015), and in both pediatric and adult patients the use of iodinated contrast media may increase doses by up to 35% (Dawson and Punwani 2009). Nevertheless, these doses are much lower—by 3–4 orders of magnitude—compared to those associated with EBRT or RAI. No publications report an increased incidence of hypothyroidism after diagnostic radiation exposure of the thyroid by CT. Perhaps the ongoing International Pediatric CT Scan study (https://epi-ct.iarc.fr/index.php; last accessed 11 April 2020) organized by the International Agency for Research on Cancer may allow new insights.

### Atomic bombing survivors

Imaizumi et al. (2017) summarized the current knowledge about thyroid dysfunction and autoimmune thyroid diseases among atomic bomb survivors from Hiroshima and Nagasaki, focusing on those who were children age < 10 years at the time of exposure (n = 2,668) as the most vulnerable age subgroup. Mean age at the examination of these survivors was 68.2 ± 2.7 years. Thyroid radiation doses ranged from 0–4.0 Gy, with a mean of 0.182 Gy and a median of 0.018 Gy. Hypothyroidism (sublinical or overt) was detected in 4.8% (129/2,668) of the exposed people. Of the patients with overt hypothyroidism, 44% (57/129; 2.1% of the overall cohort) were thyroid autoantibody-positive and 56% (72/129; 2.7% of the overall cohort), thyroid autoantibody-negative. Additionally, 21.5% (573/2,668) of the subgroup was antibody-positive without thyroid dysfunction. Most importantly, no significant association of TSH concentration or antibody detectability with thyroid radiation dose was found.

### People exposed to nuclear reactor accidents

In 1986, it was revealed that during the initial years of plutonium production at the Hanford Nuclear Site in the US (1944–1957), large amounts of gaseous and vaporized radionuclides—among them I-131—were released into the atmosphere (Davis et al. 2004). The Hanford Thyroid Disease Study (HTDS) reconstructed thyroid doses for 3440 individuals exposed to these discharges, which were estimated at between 0.003 and 2823 mGy (mean: 174 mGy, median: 97 mGy). More than 60% of the subjects were children age < 10 years during the period of radioactive releases. For subclinical or overt cases of hypothyroidism in people exposed to the releases, Davis et al. (2004) reported cumulative incidences of 11.7% in women and 3.7% in men. The cumulative incidence of TPOAb positivity amounted to 20.8% in females and 10.2% in males. Neither the incidence of subclinical and overt hypothyroidism (*P* = 0.61) nor that of autoimmune thyroiditis (*P* = 0.91), was found to have a significant statistical correlation with the radiation dose to the thyroid (Davies et al. 2004).

After the Chernobyl accident of 1986, hypothyroidism was detected in 4/83 (4.8%) of the emergency workers with the highest radiation exposure among those treated for acute radiation syndrome at Burnasyan Federal Medical Biophysical Center of the Federal Medical Biological Agency in Moscow. Hypothyroidism developed during the first 5 years in 3 of the 4 cases, developing later in 1 case. The highest thyroid dose reported in these 4 patients amounted to 11 Gy, however, thyroid doses for the whole cohort of 83 ARS patients were not available (UNSCEAR 2011).

**Fig. 4:**
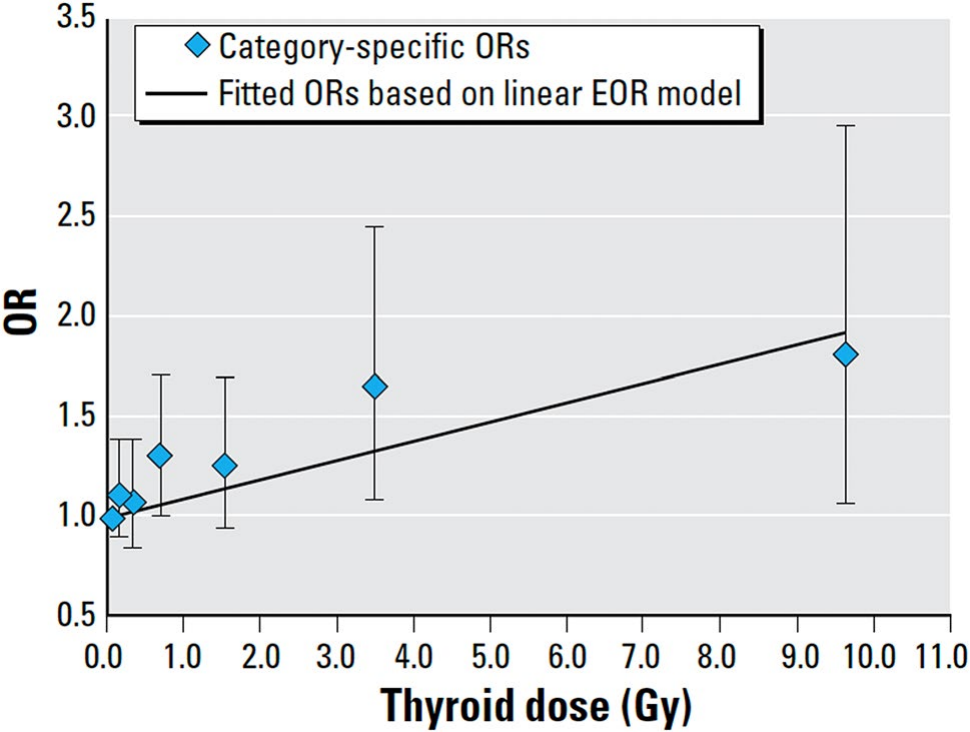
Association between prevalence of hypothyroidism and estimated radiation dose to the thyroid: Ukrainian–American cohort study of thyroid cancer and other thyroid diseases after the Chernobyl accident, 1998–2000. The dose–response line was adjusted to pass through the lowest thyroid dose category. Data points represent the thryoid dose category-specific odds ratios (ORs) with 95% CIs. The line represents fitted ORs based on a linear EOR model (Ostroumova et al. 2009, with permission)

Oustroumova et al. investigated subjects from the Ukraine (*N* = 11,853) (Ostroumova et al. 2009) or Belarus (*N* = 10,827) (Ostroumova et al. 2013) who were children and adolescents < 18 years old at the time of the Chernobyl accident. These authors described significant associations between the presence of subclinical hypothyroidism, defined as elevated TSH levels (> 4 mU/L) and thyroid hormone levels within the reference range, and cumulative thyroid radiation doses in both cohorts. Thyroid doses in the Ukrainian cohort (Ostroumova et al. 2009) covered a broad range, from 0 to 40.8 Gy, with a mean of 0.79 Gy. Among 11,853 individuals included in the analysis, 719 (6.1%) had subclinical hypothyroidism, and 14 (0.1%), overt hypothyroidism with decreased levels (< 10 pmol/L) of serum-free thyroxine. The risk for subclinical hypothyroidism decreased with age at the examination and was similar for males and females. The study revealed a small association between I-131 thyroid doses and subclinical hypothyroidism that was best described with a linear model (Fig. 4), with the excess odds ratio (EOR) of hypothyroidism of 0.10 (95% CI, 0.03–0.21) per Gy.

**Fig. 5:**
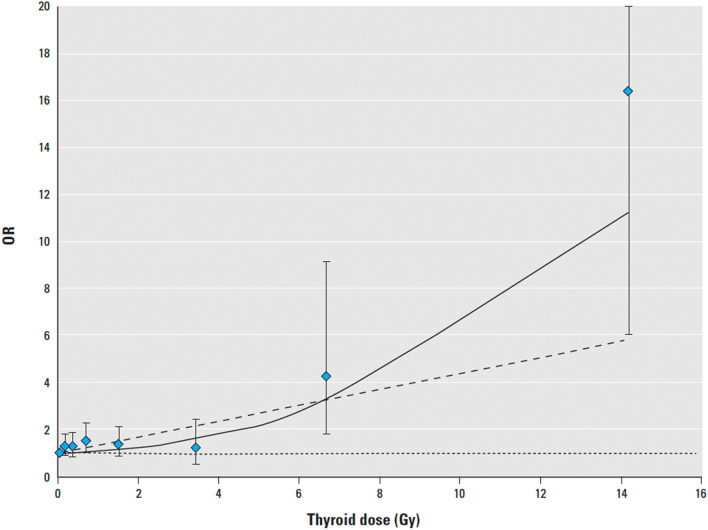
Dose–response association between prevalence of hypothyroidism and estimated thyroid from I-131 in a cohort study of thyroid cancer and other thyroid diseases after the Chernobyl accident in Belarus, 1996–2003. The dose–response line was adjusted to pass through the lowest dose category. The data points represent dose category-specific ORs with 95% CIs (whiskers). Curves represent fitted ORs based on linear (dotted line) and linear-quadratic (solid line) EOR models (Ostroumova et al. 2013, with permssion)

In the cohort from Belarus with 10,827 subjects < 18 years old at the time of the Chernobyl accident (Ostroumova et al. 2013), thyroid doses covered a broad range too, from 0 to 26.6 Gy, with a mean of 0.54 Gy. In 301 subjects (2.8%), subclinical hypothyroidism was detected, and in 18 (0.2%), overt hypothyroidism. The risk for hypothyroidism decreased with increasing age at exposure or examination and was similar for males and females. The study revealed an association between increasing thyroid doses from I-131 and greater risk of subclinical hypothyroidism, with an EOR per Gy for this condition of 0.34 (95% CI, 0.15–0.62). This EOR is higher than that in the Ukrainian cohort (Ostroumova et al. 2009), and the effect/risk relationship in the Belarussian cohort is better described with a linear-quadratic model (Fig. 5). TPOAb were found in 5.7% of the subjects investigated, with no positive association of the antibodies’ presence with the radiation dose.

In a study from Belarus, Drozd et al. (2003) screened a cohort of children in the Khoiniki region of the Gomel oblast who were exposed to Chernobyl fallout in utero (*n* = 100) or during the first or second years of life (*n* = 228). The lowest average thyroid dose in children exposed in utero was 360 mGy. The highest average thyroid dose, 1.1 Gy, was registered in children exposed at 0–1.5 years of age receiving mixed nutrition (breastfeeding plus cow milk consumption). Among children exposed in utero, those irradiated in the first trimester of gestation received the lowest average thyroid dose, close to zero, which is easy to explain, because the thyroid starts to metabolize iodine no earlier than at the end of the first trimester (Berkowski et al. 2003). Interestingly, compared with those exposed in the third trimester of gestation, children exposed in the first trimester reportedly had significantly smaller average thyroid volumes, and a higher average TSH level (2.8 ± 1.44 vs. 2.3 ± 1.02 mU/L). In parallel, higher percentages of thyroglobulin autoantibody positivity and TPOAb positivity were found in the group of children exposed in utero in the first trimester versus in the third trimester. However, the subgroups compared are small, with 20 and 36 subjects, respectively. On the other hand, Drozd et al. (2010) could reproduce in a separate sample of children irradiated in the Chernobyl accident, from the Stolin region of the Brest oblast (*n* = 3311), the observation of smaller thyroid volumes and higher TSH levels in those exposed during the first trimester in utero than in those exposed during the third trimester.

Pacini et al. (1998, 2002) studied the prevalence of thyroid autoantibodies in two groups of children and adolescents from Belarus exposed to Chernobyl fallout: those in the Khoiniki region (*n* = 287) or those in a non-contaminated region, Braslav (*n* = 208). In a study published in 1998, the proportion of TPOAb–positive subjects was much higher in Khoiniki versus Braslav: 19.5% vs. 3.8% (*P* < 0.0001). Ten years later, a consortium with Pacini as a co-author reassessed the effects of Chernobyl-related radiation exposure on thyroid autoimmunity in 1433 serum samples from subjects who were 13–17-year-old adolescents at the time of the reactor accident, living in contaminated and non-contaminated villages of Ukraine, Belarus, or Russia (Agate et al. 2008). TPOAb prevalence was still higher in contaminated than in noncontaminated Belarusian villages (6.4% vs. 2.4%, *P* = 0.02) but lower than reported a decade earlier in different contaminated Belarusian villages. So the increased frequency of TPOAb positivity was less evident than was previously reported and was not accompanied by thyroid dysfunction. No differences in TPOAb prevalence were found between contaminated versus non-contaminated Ukrainian or Russian villages (Agate et al. 2008).

The Fukushima Daiichi accident in 2011 released much less radioactivity (appr. 10%) as compared to the Chernobyl accident in 1986 (UNSCEAR 2015), and thyroid doses to the population were much lower due to immediate withdrawal of contaminated food and milk and evacuation from highly exposed regions. Yamashita et al. (2018) summarized the results of the first and second rounds of the large-scale ultrasound survey after the Fukushima accident, demonstrating a high detection rate of thyroid cancer in children and young adolescents by a mass screening effect. There appeared to be no association between the incidence of childhood thyroid cancer and external radiation dose after the accident (Ohira 2018). Mean thyroid doses in children investigated in this Fukushima Health Management Survey were < 5 mGy whereas mean doses ranged between 300 mGy and 1 Gy in children from Russia, the Ukraine, and Belarus after the Chernobyl accident (Nagataki 2016). A recent study (Kim 2020) confirmed that individual thyroid-equivalent doses to Fukushima residents just after the accident were < 30 mSv. Not surprisingly, Watanobe et al. (2015) reported no indication of excess hypothyroidism, autoimmune thyroiditis, or other thyroid dysfunction among 1137 children and adolescents from the Fukushima Prefecture who were age < 18 years at the time of the accident (UNSCEAR 2015).

Nevertheless, thyroid doses for 12 employees working at the Fukushima-Daiichi plant on March 11th 2011 were really high, ranging between 2 and 12 Gy (UNSCEAR 2015). Despite that, medical follow-up for the first 5 years after exposure did not reveal any thyroid abnormalities such as hypothyroidism or autoimmune thyroiditis (Akashi M., presentation, 4th Wuerzburg-Moscow Seminar “Radiation Emergency Medicine”, Würzburg, Germany 16–17 June 2016).

## Discussion

In the general population, subclinical hypothyroidism with TSH blood levels elevated above 4 mU/L seems not to be rare, with prevalences ranging between 2 and 5% in Germany and the US (Hollowell et al. 2002; Chaker et al. 2017; Khattak et al. 2016). However, it should be taken into account that the upper TSH reference level usually is defined as the 97.5th percentile of the distribution of “normal” healthy subjects. This means that 2.5% of TSH levels of healthy people will exceed 4 mU/L just for statistical reasons. Hence a considerable proportion of subjects reported in the literature as being subclinically hypothyroid are in reality euthyroid. Beyond that, it must be borne in mind that the upper level of normal for TSH seems to be more adequately defined as 4.5 mU/L for 7-year-old children and 5.0 mU/L for 15-year-old children (Taylor et al. 2017). On the other hand, overt hypothyroidism with elevated TSH (usually > 10 mU/L), decreased blood levels of the thyroid hormones T4 and T3, and with clinical symptoms, is usually found in < 2% of the general population (Chaker et al. 2017). Hypothyroidism is more frequent in regions with nutritional iodine deficiency, e.g., large parts of Africa and Asia (Chaker et al. 2017), since iodine is a component of the T4 and T3.

However, the most frequent cause of hypothyroidism is autoimmune thyroiditis, when autoantibodies against cellular components of the thyroid induce destruction of the gland’s tissue. In the US, TPOAb are measurable in 8.0% of males and 14.6% of females (Hollowell et al. 2002). Compared to the US, where the iodine supply is sufficient, Germany remains a country with moderate iodine deficiency, and TPOAb are detectable in 3.7% of males and 7.1% of females (Khattak et al. 2016). So it is understandable, that sublinical hypothyroidism is more prevalent in the USA with 5% overall, as compared to Germany, with 2% overall (Hollowell et al. 2002; Khattak et al. 2016). Generally, hypothyroidism and autoimmune thyroiditis in the general population are more frequently encountered in females than in males, and these conditions’ incidence increases with age.

Radiation can destroy thyroid tissue with consequent hypothyroidism and, besides this mechanism, radiation may lead to hypothyroidism indirectly, by induction of autoimmune thyroiditis. Many years ago already, Malone and McCullen (1976) described tissue destruction as an early “deterministic” radiation effect with threshold-dependency and dose-dependency. Additionally, they assumed that a second, late effect without dose threshold might be caused by induction of thyroid autoimmunity. However, it is difficult to separate these radiation effects for several reasons.

One such reason relates to Graves’ disease, which is treated with high activities of radioiodine (I-131) leading to thyroid doses of 200–400 Gy (Peters et al.^1997^). Compared to routine practice with EBRT, and notwithstanding expert recommendations (Häenscheid et al. 2013), thyroid dosimetry regrettably is performed only rarely in RAI. Therefore, in many published papers, it is not possible to relate rates of hypothyroidism to thyroid doses (in Gy). However, it can be concluded from a prospective study in Germany (Peters et al. 1997) that after approximately 6 months, no remarkable induction of hypothyroidism occurs below a mean thyroid dose of 50 Gy, whereas 50% of patients with Graves´ disease become hypothyroid at a dose of 220 Gy, and 80% at 400 Gy (Fig. 1). The other reason why patients with Graves´ disease cannot be compared with other populations medically or accidentally exposed to radiation is the auto-immunogenic nature of Graves´ disease itself. Pathophysiologically, autoantibodies binding to the TSH receptor stimulate overproduction of thyroid hormones. Typically this process is accompanied by overactivity of TPOAb (Peters et al. 1997), which, after years, may induce hypothyroidism as the natural course in 10–20% of patients with Graves’ disease.

As alluded to above, compared to RAI, where dosimetric data are sparse, precise dosimetry is standard in EBRT. Surprisingly, hypothyroidism appears to develop at much lower thyroid doses after EBRT of the neck; 50% of patients given 45 Gy and 80% subjected to 55 Gy become hypothyroid. Hypothyroidism rarely develops in patients whose thyroids were irradiated with < 10 Gy (Fig. 2). The risk for hypothyroidism is inversely associated with age at exposure (Rønjom et al. 2015; Inskip et al. 2018), and TSH levels start to increase 6 months after neck irradiation, 80% of patients becoming hypothyroid in the first 5 years of follow-up (Rønjom 2016).

There are several reasons why lower thyroid doses of EBRT induce hypothyroidism with comparable frequency as do 4–fivefold higher doses of “internal radiotherapy” with I-131. There is evidence from studies in children (Inskip et al. 2018), that in patients treated with EBRT, hypothyroidism may be induced directly by irradiation of the thyroid and indirectly too, if the pituitary, which controls the thyroid, is in the radiation field (e.g., in tumors of the skull base). Another reason may be that ß-particles of I-131 taken up by thyroid cells have a short range of only 0.4 mm in tissue, so that blood vessels supplying the thyroid are out of range, which is not the case in EBRT, where structures adjacent to the thyroid also are irradiated. The early literature (Doniach 1957; Kneeland Frantz V et al. 1957) contains reports of animal studies comparing the effects of internal irradiation by I-131 with external X-ray exposure. However, the authors mainly were interested in the induction of tumors and not of hypothyroidism, and a real comparison of the effects of both types of therapy was not possible because of the weakness of internal dosimetric techniques at that time (Ron & Brenner 2010; Hänscheid et al. 2013).

Radiation from medical diagnostics with doses of 10–30 mGy, which may reach up to 50 mGy in children, seem to be too low to induce hypothyroidism. However, systematic studies about risk for this condition in children are sparse.

In A-bomb survivors, thyroid doses were higher (mean: 182 mGy), and inhomogenously distributed (median: 18 mGy), with only a few subjects receiving doses up to 4 Gy (Imaizumi et al. 2017). Overt or subclinical hypothyroidism was found in 4.8% of the people exposed, but no association with radiation doses was described. Interestingly, the rate of A-bomb survivors showing elevated titers of TPOAb was high, at 21.5%, but again, no correlation with thyroid doses was found (Imaizumi et al. 2017). Compared to prevalence in other countries, the rates of subclinical hypothyroidism and TPOAb positivity in the general population are high in Japan, which can be explained by abundant nutritional iodine supply.

Thyroid doses after the accidental releases of I-131 at the Hanford Nuclear Site (Davies et al. 2004) were in a similar range as in A-bomb survivors (mean: 174 mGy) but more homogeneously distributed (median: 97 mGy). Davis et al. (2004) reported for subclinical or overt cases of hypothyroidism a comparable overall incidence (7.8.%) to that in A-bomb survivors (4.8%) (Imaizumi et al. 2017), and a lower incidence of TPOAb positivity (15.7% vs. 31.5%), but here too, no correlation with thyroid radiation doses.

Conversely, thyroid doses after the Chernobyl accident cover a broad range, with relatively high levels in those exposed as children and adolescents. The exposure ranged from 0 to 40.8 Gy with a mean of 0.79 Gy in Ukraine, and from 0 to 26.6 Gy with a mean of 0.54 Gy in Belarus (Ostroumova et al. 2009, 2013). Unfortunately, the surrounding population was not informed about the meltdown of the Chernobyl reactor at the time of the accident or advised to discard contaminated food and milk. These circumstances mostly explain the steep increase of thyroid cancer incidence, especially in young children, starting 4–5 years after the accident (UNSCEAR 2011). With respect to thyroid dysfunction, Ostroumova et al. (2009, 2013) described an association of subclinical hypothyroidism with thyroid radiation doses and calculated mean excess observed risks per Gy of 0.1 for the Ukraine and 0.3 for Belarus. Generally, the observed risks are small, and Figs. 4 and 5 show that significant increases of these risks were observed only after thyroid doses higher than 3 Gy, so that extrapolation to lower doses seems not to be justified. There are some other findings which remain unclear, e.g., the higher risk in Belarus despite lower doses than measured in Ukraine, and the decreasing risk for hypothyroidism with increasing age at exposure in Belarus, but no such effect in Ukraine. Here confounders such as, e.g., nutritional iodine supply or so-called endocrine disruptors e.g., nitrates in food and drinking water, may play a role (Drozd et al. 2018). An argument for the latter hypothesis may be that in Belarus, the EOR for hypothyroidism was higher in rural than in urban residents.

After the Fukushima Daiichi accident, mean thyroid doses in children were < 30 mGy (Nagataki 2016; Kim 2020). Unsurprisingly, no excess frequency of hypothyroidism, autoimmune thyroiditis, or other thyroid dysfunction was detected in the Fukushima Health Mangement Survey (Watanobe et al., 2015). On the other hand, thyroid doses for 12 employees working on the Fukushima-Daiichi plant on 11 March 2011 were really high, ranging between 2 and 12 Gy (UNSCEAR 2015). Nonetheless, medical follow-up of these employees for the first 5 years after exposure did not reveal any thyroid abnormalities such as hypothyroidism or autoimmune thyroiditis (Akashi 2016).

## Conclusions—open questions

Historically, hypothyroidism after external irradiation of the thyroid in adulthood was considered not to develop below a dose threshold of 10–20 Gy (Clifton 1986; Ron & Brenner 2010). More recent data from patients receiving EBRT suggest a dose threshold for an increased risk of hypothyroidism of 10 Gy (Vogelius et al. 2011). Data from patients with Graves´disease after I-131 “internal radiotherapy” indicate that hypothyroidism rarely occurs below thyroid doses of 50 Gy (Peters et al. 1997). This observation corresponds to data from highly exposed workers of the Fukushima plant, who tolerated a thyroid dose of up to 12 Gy without developing hypothyroidism, at least during the first 5 years of follow-up (Akashi 2016). On the other hand, experiences in children exposed to Chernobyl fallout as well as to contaminated food and milk indicate that the dose threshold for increased risk of hypothyroidism may be considerably lower, i.e., 3–5 Gy (Ostrumova et al. 2009, 2013). This finding has been confirmed by Imaizumi et al. (2017), who stated that Ostroumova’s (2009, 2013) findings align with results in young A-bomb survivors age < 10 years at the time of exposure, who showed no significant dose–response for hypothyroidism below a thyroid dose of 4 Gy.

Regarding thyroid autoimmunity after radiation exposure, there seems to be clear evidence from mechanistic models that this condition may develop due to antibody production after the presentation of antigenic material from necrotic thyroid tissue (Riley et al. 2019). However, data from different exposure scenarios are inconsistent, and sociodemographic factors and confounders such as nutritional iodine supply and nitrate exposure may play an important role. Up to now, no data are available about autoimmune thyroiditis after radiation exposure and its possible association with depression and anxiety disorders. However, the high prevalences of depression and suicide observed after the Fukushima accident (Maeda and Oe 2017) seem to be related to the massive posttraumatic stress and not to thyroid disturbances.

Generally, the evidence about radiation induction of hypothyroidism is based mainly on follow-up studies in medically-exposed patients treated with EBRT or RAI and epidemiological studies in populations exposed to radiation from A-bombs or nuclear accidents. To date, systematic studies about the underlying mechanisms are weak or absent. So important questions still remain to be answered about the development of hypothyroidism and thyroid hypoplasia after radiation exposure, e.g*.*, in utero, about the interaction between autoimmunity and hypofunction, and about the apparently different effects of internal versus external irradiation.

In any case, follow-up of thyroid function after EBRT or RAI is mandatory and patients with hypothyroidism needing thyroid hormone replacement will not be overlooked. In populations exposed to accidental radiation, a conservative threshold dose of 3–5 Gy for the most vulnerable group of children and adolescents is proposed, which should be implemented in emergency management plans including long-term follow-up of this group.

## References

[CR1] Agate L, Mariotti S, Elisei R, Mossa P, Pacini F, Molinaro E, Grasso L, Masserini L, Mokhort T, Vorontsova T, Arynchyn A, Tronko MD, Tsyb A, Feldt-Rasmussen U, Juul A, Pinchera A (2008). Thyroid autoantibodies and thyroid function in subjects exposed to Chernobyl fallout during childhood: evidence for a transient radiation-induced elevation of serum thyroid antibodies without an increase in thyroid autoimmune disease. J Clin Endocrinol Metab.

[CR2] Akashi M (2016) Presentation 4th Wuerzburg-Moscow Seminar “Radiation Emergency Medicine”, Wuerzburg 16./17.6.2016

[CR3] Berkovski V, Eckerman KF, Phipps AW, Nosske D (2003). Dosimetry of radioiodine for embryo and fetus. Radiat Prot Dosimetry.

[CR4] Bonnema SJ, Hegedüs L (2012). Radioiodine therapy in benign thyroid diseases: effects, side effects, and factors affecting therapeutic outcome. Endocr Rev.

[CR5] Chaker L, Bianco AC, Jonklaas J, Peeters RP (2017). Hypothyroidism. Lancet.

[CR6] Clifton KH (1986). Thyroid and mammary radiobiology: radiogenic damage to glandular tissue. Br J Cancer Suppl.

[CR7] Davis S, Kopecky KJ, Hamilton TE, Onstad L, the Hanford Thyroid Disease Study Team (2004). Thyroid neoplasia, autoimmune thyroiditis, and hypothyroidism in persons exposed to iodine 131 from the Hanford Nuclear Site. JAMA.

[CR8] Dawson P, Punwani S (2009). The thyroid dose burden in medical imaging: a re-examination. Eur J Radiol.

[CR9] Doniach I (1957). Comparison of the carcinogenic effect of x-irradiation with radioactive iodine on the rat's thyroid. Brit J Cancer.

[CR10] Drozd V, Danilova L, Lushchyk M, Leonova T, Platonova T, Grigorovich A, Sivuda I, Branovan I, Biko J, Reiners C (2010) Detection of increased frequency of thyroid hypoplasia in subjects irradiated in utero as the result of Chernobyl catastrophe. International Thyroid Cancer Congress Paris, https://b-com.mci-group.com/Abstract/Statistics/AbstractStatisticsViewPage.aspx?AbstractID=31150

[CR11] Drozd V, Mityukova T, Bazylchik S, Davidova E, Gavrilin Y, Khrusch V, Shinkarev S, Reiners C, Biko J (2003). Screening of thyroid status in children exposed to ionizing radiation in utero and at the first year of life as a result of the Chernobyl accident. Int J Radiat Med.

[CR12] Drozd VM, Branovan I, Shiglik N, Biko J, Reiners C (2018). thyroid cancer induction: nitrates as independent risk factors or risk modulators after radiation exposure, with a focus on the Chernobyl accident. Eur Thyroid J.

[CR13] Dunkelmann S, Wolf R, Koch A, Kittner C, Groth P, Schuemichen C (2004). Incidence of radiation-induced Graves' disease in patients treated with radioiodine for thyroid autonomy before and after introduction of a high-sensitivity TSH receptor antibody assay. Eur J Nucl Med Mol Imaging.

[CR14] Hänscheid H, Canzi C, Eschner W, Flux G, Luster M, Strigari L, Lassmann M (2013). EANM Dosimetry Committee series on standard operational procedures for pre-therapeutic dosimetry II. Dosimetry prior to radioiodine therapy of benign thyroid diseases. Eur J Nucl Med Mol Imaging.

[CR15] Hollowell JG, Staehling NW, Flanders WD, Hannon WH, Gunter EW, Spencer CA, Braverman LE (2002). Serum TSH, T(4), and thyroid antibodies in the United States population (1988 to 1994): National Health and Nutrition Examination Survey (NHANES III). J Clin Endocrinol Metab.

[CR16] Imaizumi M, Ohishi W, Nakashima E, Sera N, Neriishi K, Yamada M, Tatsukawa Y, Takahashi I, Fujiwara S, Sugino K, Ando T, Usa T, Kawakami A, Akahoshi M, Hida A (2017). Thyroid dysfunction and autoimmune thyroid diseases among atomic bomb survivors exposed in childhood. J Clin Endocrinol Metab.

[CR17] Inskip PD, Veiga LHS, Brenner AV, Sigurdson AJ, Ostroumova E, Chow EJ, Stovall M, Smith SA, Weathers RE, Leisenring W, Robison LL, Armstrong GT, Sklar CA, Lubin JH (2018). Hypothyroidism after radiation therapy for childhood cancer: a report from the childhood cancer survivor study. Radiat Res.

[CR18] International Agency for Research on Cancer: EPI-CT: International Pediatric CT Scan Study. WHO Lyon https://epi-ct.iarc.fr/index.php

[CR19] Khattak RM, Ittermann T, Nauck M, Below H, Völzke H (2016). Monitoring the prevalence of thyroid disorders in the adult population of Northeast Germany. Popul Health Metr.

[CR20] Kim E, Yajima K, Hashimoto S, Tani K, Igarashi Y, Iimoto T, Shigure N, Tatsuzaki H, Akashi M, Kurihara O (2020). Reassessment of thyroid doses to 1,080 children examined in a screening survey after the 2011 Fukushima nuclear disaster. Health Phys.

[CR21] Kneeland Frantz V, Morton F, Mm K, Wa H, Me P, Quimby Eh (1957). A comparison of the carcinogenic effect of internal and external irradiation on the thyroid gland of the male Long-Evans rat. Endocrinology.

[CR22] Lin Z, Yang Z, He B, Wang D, Gao X, Tam SY, Wu VWC (2018). Pattern of radiation-induced thyroid gland changes in nasopharyngeal carcinoma patients in 48 months after radiotherapy. PLoS ONE.

[CR23] Maeda M, Oe M (2017). Mental health consequences and social issues after the Fukushima disaster. Asia Pac J Public Health.

[CR24] Metso S, Jaatinen P, Huhtala H, Luukkaala T, Oksala H, Salmi J (2004). Long-term follow-up study of radioiodine treatment of hyperthyroidism. Clin Endocrinol (Oxf).

[CR25] Malone JF, Cullen MJ (1976). Two mechanisms for hypothyroidism after 131I therapy. Lancet.

[CR26] Nagataki S (2016). minimizing the health effects of the health effects of the reactor accident in Fukushima on thyroids. Eur J Thyroid.

[CR27] Nagayama Y (2018). Thyroid autoimmunity and thyroid cancer—the pathogenic connection: a 2018 update. Horm Metab Res.

[CR28] Ohira T, Takahashi S, Ohtsuru A, Midorikawa S, Sa S, Matsuzukawa T, Shimura H, Ishikawa T, Sakai A, Yamashita S, Tanigawa K, Ohto H, Kamiya K, Suzuki Sh (2018). Associations between childhood thyroid cancer and external radiation dose after the Fukushima Daiichi nuclear power plant accident. Epidemiol.

[CR29] Ostroumova E, Brenner A, Oliynyk V, McConnell R, Robbins J, Terekhova G, Zablotska L, Likhtarev I, Bouville A, Shpak V, Markov V, Masnyk I, Ron E, Tronko M, Hatch M (2009). Subclinical hypothyroidism after radioiodine exposure: Ukrainian-American cohort study of thyroid cancer and other thyroid diseases after the Chornobyl accident (1998–2000). Environ Health Perspect.

[CR30] Ostroumova E, Rozhko A, Hatch M, Furukawa K, Polyanskaya O, McConnell RJ, Nadyrov E, Petrenko S, Romanov G, Yauseyenka V, Drozdovitch V, Minenko V, Prokopovich A, Savasteeva I, Zablotska LB, Mabuchi K, Brenner AV (2013). Measures of thyroid function among Belarusian children and adolescents exposed to iodine-131 from the accident at the Chernobyl nuclear plant. Environ Health Perspect.

[CR31] Pacini F, Agate L, Molinari E, Elisei R, Pinchera A (2002). Thyroid diseases around Chernobyl: from autoimmune diseases to malignant tumors. Int Congr Ser.

[CR32] Pacini F, Vorontsova T, Molinaro E, Kuchinskaya E, Agate L, Shavrova E, Astachova L, Chiovato L, Pinchera A (1998). Prevalence of thyroid autoantibodies in children and adolescents from Belarus exposed to the Chernobyl radioactive fallout. Lancet.

[CR33] Peters H, Fischer C, Bogner U, Reiners C, Schleusener H (1997). Treatment of Graves' hyperthyroidism with radioiodine: results of a prospective randomized study. Thyroid.

[CR34] Riley AS, McKenzie GAG, Green V, Schettino G, England RJA, Greenman J (2019). The effect of radioiodine treatment on the diseased thyroid gland. Int J Radiat Biol.

[CR35] Rodondi N, den Elzen WPJ, Bauer DC, et al. for the Thyroid Studies Collaboration Study Group (2010) Subclinical hypothyroidism and the risk of coronary heart disease and mortality. JAMA 304:1365–74. 10.1001/jama.2010.1361 PMC3923470 20858880

[CR36] Ron E, Brenner A (2010). Non-malignant thyroid diseases after a wide range of radiation exposures. Radiat Res.

[CR37] Rønjom MF (2016). Radiation-induced hypothyroidism after treatment of head and neck cancer. PhD Thesis. Dan Med J.

[CR38] Rønjom MF, Brink C, Bentzen SM, Hegedüs L, Overgaard J, Petersen JB, Primdahl H, Johansen J (2015). External validation of a normal tissue complication probability model for radiation-induced hypothyroidism in an independent cohort. Acta Oncol.

[CR39] Spampinato MV, Tipnis S, Tavernier J (2015). Huda W (2015) Thyroid doses and risk to paediatric patients undergoing neck CT examinations. Eur Radiol..

[CR40] Siegmann EM, Müller HHO, Luecke C, Philipsen A, Grömer TW (2018). Association of depression and anxiety disorders with autoimmune thyroiditis. JAMA Psychiatry.

[CR41] Shinohara N, Takahashi M, Kamishima T, Ikushima H, Otsuka N, Ishizu A, Shimizu C, Kanayama H, Nonomura K (2011). The incidence and mechanism of sunitinib-induced thyroid atrophy in patients with metastatic renal cell carcinoma. Br J Cancer.

[CR42] Sinnott B, Ron E, Schneider AB (2010). Exposing the thyroid to radiation: a review of its current extent, risks, and implications. Endocr Rev.

[CR43] Su YP, Niu HW, Chen JB, Fu YH, Xiao GB, Sun QF (2014). Radiation dose in the thyroid and the thyroid cancer risk attributable to CT scans for pediatric patients in one general hospital of China. Int J Environ Res Public Health.

[CR44] Taylor PN, Sayers A, Okosieme O, Das G, Draman MS, Tabasum A, Abusahmin H, Rahman M, Stevenson K, Groom A, Northstone K, Woltersdorf W, Taylor A, Ring S, Lazarus JH, Gregory JW, Rees A, Timpson N, Dayan CMJ (2017). Maturation in Serum Thyroid Function Parameters Over Childhood and Puberty: Results of a Longitudinal Study. Clin Endocrinol Metab.

[CR45] United Nations Scientific Committee on the Effects of Atomic Radiation: Sources and effects of ionizing radiation. UNSCEAR 2008 Report to the General Assembly with Scientific Annexes. Volume II Scientific Annexes C, D and E. United Nations New York, 2011

[CR46] United Nations Scientific Committee on the Effects of Atomic Radiation: Developments since the 2013 UNSCEAR Report on the levels and effects of radiation exposure due to the nuclear accident following the Great East-Japan Earthquake and Tsunami. United Nations New York, 2015

[CR47] Vogelius IR, Bentzen SM, Maraldo MV, Petersen PM, Specht L (2011). Risk factors for radiation-induced hypothyroidism: a literature-based meta-analysis. Cancer.

[CR48] Watanobe H, Furutani T, Nihei M, Sakuma Y, Yanai R, Takahashi M, Sato H, Sagawa F (2015). The thyroid status of children and adolescents in Fukushima Prefecture examined during 20–30 months after the Fukushima nuclear power plant disaster: a cross-sectional, observational study. PLoS ONE.

[CR49] World Health Organization. (2004) ICD-10 : International statistical classification of diseases and related health problems : tenth revision, 2nd ed. World Health Organization. https://apps.who.int/iris/handle/10665/42980 3376487

[CR50] Yamashita S, Suzuki Sh, Sa S, Shimura H, Saenko V (2018). Lessons from Fukushima: latest findings of thyroid cancer after the Fuksuhima nuclear power plant accident. Thyroid.

